# Feasibility analysis of incorporating infertility into medical insurance in China

**DOI:** 10.3389/fendo.2022.967739

**Published:** 2022-09-05

**Authors:** Lin Wang, Ye Zhu, Tong Wang, Xinrong Xu, Qiuqin Tang, Jinhui Li, Yanchen Wang, Weiyue Hu, Wei Wu

**Affiliations:** ^1^ State Key Laboratory of Reproductive Medicine, Institute of Toxicology, Nanjing Medical University, Nanjing, China; ^2^ Key Laboratory of Modern Toxicology of Ministry of Education, School of Public Health, Nanjing Medical University, Nanjing, China; ^3^ Department of Obstetrics, Women’s Hospital of Nanjing Medical University, Nanjing Maternity and Child Health Care Hospital, Nanjing, China; ^4^ Department of Urology, Stanford Medical Center, Stanford, CA, United States; ^5^ National Health Commission (NHC) Key Laboratory of Neonatal Diseases, Fudan University, Children’s Hospital of Fudan University, Shanghai, China

**Keywords:** assisted reproductive technology, feasibility, *in vitro* fertilization, infertility, medical insurance

## Abstract

In recent years, the incidence of infertility has been increasing gradually, while the natural rate of population growth is declining or even at zero growth. China is observed to enter a depth of aging society, leading to more severe infertility. Infertility patients face many predicaments, and many unreasonable behaviors existed in seeking medical diagnosis and treatment, of which the main influencing factor is economic condition. In China, Beijing has taken the lead in providing medical insurance for 16 assisted reproductive technology items. Assuming that all infertile couples with the option of assisted reproduction are treated, there would be a huge market gap. The reimbursement rate can be adjusted based on some factors within the affordable range of the medical insurance fund. Progress on infertility coverage in other countries was also reviewed. This paper cited the data of medical insurance funds in China in the recent 4 years as a reference. Based on the data, it is not currently able to cover all the costs of infertility diagnosis and treatment during the research period, but it is feasible to access selective reimbursement and subsidies for those in particular need as well as to develop some commercial insurances. There is a big gap in the application of assisted reproductive technology between China and developed countries. More comprehensive and constructive policies should be formulated countrywide to standardize the market. Assisted reproduction-related technologies and acceleration of the domestic medical apparatus and instrument replacement should be improved to reduce the cost.

## Introduction

Over the past three decades, the age of first marriage for Chinese women has been rising, while the average fertility rate is falling sharply. According to the National Bureau of Statistics, the natural population growth rate in 2021 will be 0.34‰, which is lower than that of 2020 (1.45‰) and 2019 (3.34‰). Although the fertility rate has recovered after the comprehensive implementation of the two-child policy, the overall fertility rate is still far below the replacement level (1). The decline is mainly caused by two factors: lack of will and ability. Childbearing costs continue rising, which significantly reduce the social fertility willingness. At the same time, a pretty big contradiction exists between the high infertility rate and the insufficient availability of technology and equipment. The ability to increase the supply of medical services for the unfertile people with childbearing desires still required more improvements. “If the government subsidizes the treatment, it will increase the chances for more people to conceive children.” Gao Li, a deputy to the National People’s Congress, submitted a proposal to include infertility and assisted reproductive technology (ART) in the national medical insurance.

There is no doubt that the inclusion of the diagnosis of infertility and ART into medical insurance coverage would be a significant move to benefit more people. To a certain extent, it may overcome some infertile families’ urgency and then increase the fertility rate across the whole nation. Considering that socio-economic affordability is limited, it is necessary to comprehensively decide whether and how to include the diagnosis and treatment costs into medical insurance reimbursement. Although the reform of medical insurance has been completed, more investigations and policy implementations are needed to fill the gaps between the current coverage, reimbursement level, and people’s warranted needs.

## Review of existing studies

### The high incidence of infertility in China

Infertility refers to both men and women having the desire to conceive children, cohabiting for more than 12 months with normal sex life, and have no contraceptive measures without pregnancy. Female infertility is usually secondary to reproductive tract infection. Overall, 50% of infertility is due to men. Individually, 20 to 30% of infertility occurs solely due to male factors (2). Clinical investigation showed that the prevalence of infertility was climbing year by year and the patients trended to be younger, which was affected by diverse factors such as childbearing age, occupation, drug abuse, environmental pollution, sexual infection, and living habits. Li et al. conducted stratified and random sampling analyses and found that the smoking rate of male patients was 67.5%, and 69.70% of women had a history of induced abortion or spontaneous abortion (3). It is worth noticing that the causes and risk factors of male infertility are not identified at the population level despite a bunch of comprehensive research (4). Most of the current research is limited to individual institutions or a smaller sample size like specific city, district, and county investigations. Data from the 2015 China Infertility Survey Report showed that the incidence of infertility in China had reached 15%. In 2017, Zhou et al. conducted a population-based epidemiological survey of infertility in eight provinces in China and found that the prevalence rate of infertility was 15.5% among couples living together for more than 1 year and the wife was 20–49 years old, which reached 25% after excluding couples without fertility desire. Only 55% of infertile couples seek medical service (5) and about 20% of them were qualified for the treatment of ART.

According to demographic statistics, women aged 15 to 49 are generally defined as women of childbearing age. Due to the severe aging trend in the whole society of China, women aged 45 to 49 were considered the major proportion of women of childbearing age (19.7%), and women of non-optimal childbearing age (over 35 years old) accounted for 50.6%. It is predicted that the arithmetic fertility rate of women aged 15–49 in urban, town, and rural areas will continue to decline from 25.35, 33.18, and 41.27‰ in 2010 to 5.37, 18.34, and 12.03‰ in 2050, respectively (6).

### Male infertility and overall male health

It is estimated that male factors cause about 50% of infertility, but male factors rarely cause attention to couples’ infertility (7). Male factors must be fully considered in infertility treatment. According to spermatogenesis, male infertility can be divided into congenital, acquired, and idiopathic factor-related diseases (8). Age-standardized male infertility rate increased annually by 0.291% in the last three decades (9). Sperm counts have dropped over 50% in the past few decades (10, 11). Congenital factors include chromosomal abnormalities, cryptorchidism, and absence of vas deferens. Varicocele is the most common epigenetic factor, with a prevalence of 40%. Urogenital infections are also frequent. Idiopathic risk factors include exposure to tobacco, alcohol, drugs, or occupational productive toxins as well as an unhealthy diet (9). Moreover, 15% of adult men have clinical varicocele, 35% present infertility, and up to 81% may have secondary infertility (12). The infertile male should undergo timely semen analysis, physical examination, hormonal evaluation, gene testing, and imaging as well as organic surgery (7). Correcting sperm defects should be included in the management practices of infertile couples, such as severe oligospermia leading to a low success rate of intracytoplasmic mirror injection (13). At present, it is not ruled out that COVID-19 will damage the male reproductive system and lead to infertility (14).

There is growing evidence that impaired male fertility is a potential predictor of impaired male health status, but the cause of the relationship is currently unclear (15). As mentioned in the previous paragraph, male infertility deserves more attention and is becoming a significant predictive biomarker of overall male health and survival. Some scholars conducted a comprehensive study on male infertility in the past three decades, revealing that male infertility is associated with cardiovascular disease, cancer, chronic disease, and even mortality to varying degrees (16).

Male hypogonadism is a hyposexual disease caused by the reduction of androgen or the failure of androgen function, and it is very likely to cause male infertility. Clinical and subclinical hypogonadism (16, 17) is associated with morbidity and mortality from cardiovascular diseases. Male infertility may be an independent predictor of cardiovascular diseases (18), such as hypertension, heart disease, and peripheral vascular disease. The incidence of cardiovascular disorders in infertile men, especially varicocele patients, is higher than that in men having children (19). The infertile male patients have been found to have more comorbidities like diabetes mellitus, pulmonary diseases (chronic bronchitis, emphysema, allergic bronchitis, asthmatic bronchitis, and so on), connective tissue disorders (systemic lupus erythematosus, rheumatoid arthritis, and so on), peptic ulcer, and liver diseases (chronic hepatitis, cirrhosis of liver, and alcoholic cirrhosis) than fertile male (20). Male infertility can be a predictor of chronic comorbidity diseases (21). Charlson comorbidity index can evaluate the comorbidity burdens utilizing integration (22). The coexistence of comorbidity may be attributed to the common mechanism with male infertility, or the comorbidity harms a patient’s fertility directly (21). In retrospective studies, infertile men, especially those with azoospermia, are at a high risk of cancer—including a proven risk of non-Hodgkin’s lymphoma and a well-studied risk of testicular cancer (16, 23). Adenocarcinoma is very controversial in the correlation between male infertility and future cancer risk (23, 24). Genetic alterations such as disruption of the MLH1 gene may account for the potential association (25). Men with azoospermia had the highest risk of death among infertile men (26). When more than one semen parameters were abnormal, the risk of death was more than twofold (27).

### Effects of semen parameters on assisted reproduction outcomes

Semen parameters include semen volume, quality, density, motility, morphology, biochemical analysis, cell count, anti-sperm antibody testing, *etc.* Abnormal semen parameters such as sperm without acrosomes or positive anti-sperm antibodies will lead to male infertility under natural conditions (28). The semen’s concentration, motility, or morphology may have no association with the pregnancy rate of ART according to present investigations. To some extent, it may mean that ART overcomes impaired semen quality. However, whether semen parameters can affect other ART outcomes, such as fertilization, implantation, live birth, and perinatal health, is calling for further research in the administration of ART (29). A study conducted in China revealed that even if morphologically normal sperm was less than 4%, the ART clinical outcomes still stayed, while the fertilization success rate fell (30). A comprehensive study utilizing meta-analyses on the recent 20 years of teratozoospermia found no correlation between sperm morphology and pregnancy rate. However, case studies of oligozoospermia and asthenospermia found that the sperm concentration’s decrease or asthenospermia severity’s increase caused the corresponding pregnancy rates to decrease (29, 31). The meta-analysis showed that implantation rate, pregnancy rate, and live birth rate were influenced negatively by sperm DNA damage in *in vitro* fertilization (IVF) and intracytoplasmic sperm injection (ICSI) cycles (32). However, the pregnancy rate was not significant after bias adjustment. At present, studies on the effect of semen quality on ICSI are less than those on IVF, and some studies show that ICSI is more able to overcome sperm damage and achieve a successful pregnancy (29, 32).

### Infertile families faced with predicaments and psychological pressure from the low level of medical security

Infertility greatly bothered the related couples and their families—for example, due to the traditional cultural influence of blood inheritance, women are relatively under more tremendous pressure to bear children. Infertility treatment might sometimes gain some adverse influences on the relationship between husband and wife, which is embodied in “decreased sexual quality of husband and wife” (27.4%) and “couples often have conflicts” (24.1%) as well as “physical or verbal violence by her husband” (4.5%) (33). Additionally, long-term and complex treatments could also affect the harmony between couples, leading to the destruction of family stability.

The study conducted by Li et al., based on the fertility-related stress scale, indicated that infertility-related examination, IVF, and *in vitro* fertilization embryo transfer (IVF-ET) treatment would increase the stress levels of patients. In particular, the uncertainty of the treatment outcome increased the psychological burden for the patients (34). Batool Rashidi et al. investigated patients who were planning to undergo IVF or ICSI treatment by the SF-36 questionnaire and found that women scored lower than men as women were more frequently blamed for infertility (35). As a result, the complicated examination and treatment process forced more female patients to quit or pause their jobs, and the increased economic burden also induced psychological pressure. Wang et al. showed that anxiety and depression were detected in 45.68 and 50.62% of the patients with advanced age. In addition, some socio-demographic characteristics (*e*.*g*., educational level) were argued to be related to infertile women’s health (36). These negative emotions significantly influenced the quality of life and reproductive outcome (37). Yu et al. found that the greater the cost to the patients’ own time and career, the greater their anxiety (38).

### Infertility patients’ unreasonable behaviors in seeking medical diagnosis and treatment

Lv et al. showed that 42% of infertility patients never went to the hospital. The top reason for not seeking treatment in hospitals was financial status, which accounted for 23% (39). An investigation showed that standardization of examination and treatment costs can encourage infertile men to deal with stress (7). The survey by Wang et al. identified that 92.4% of patients continued their treatment with 39.7% success of pregnancy, and 55.1% patients visited two or three hospitals with 47.1% success of pregnancy. Only 70% continued the treatment with the same doctor (40). A study also found that men who voluntarily received infertility treatment could downgrade the intensity of intervention that couples need (41). It is reported that among 18–27% of infertility couples, the male partner did not undergo an infertility evaluation (42). In rural areas, patients may tend to be credulous about unscientific methods with unsystematic treatments. Moreover, income is considered to be an important determinant of access to healthcare, especially in countries that lack universal health insurance and relied mainly on private health insurance. Clinical studies have found that patients without health insurance had fewer hospital days and received more inappropriate treatments (43).

### Cost of ART and medical insurance policies

**Figure 1:**
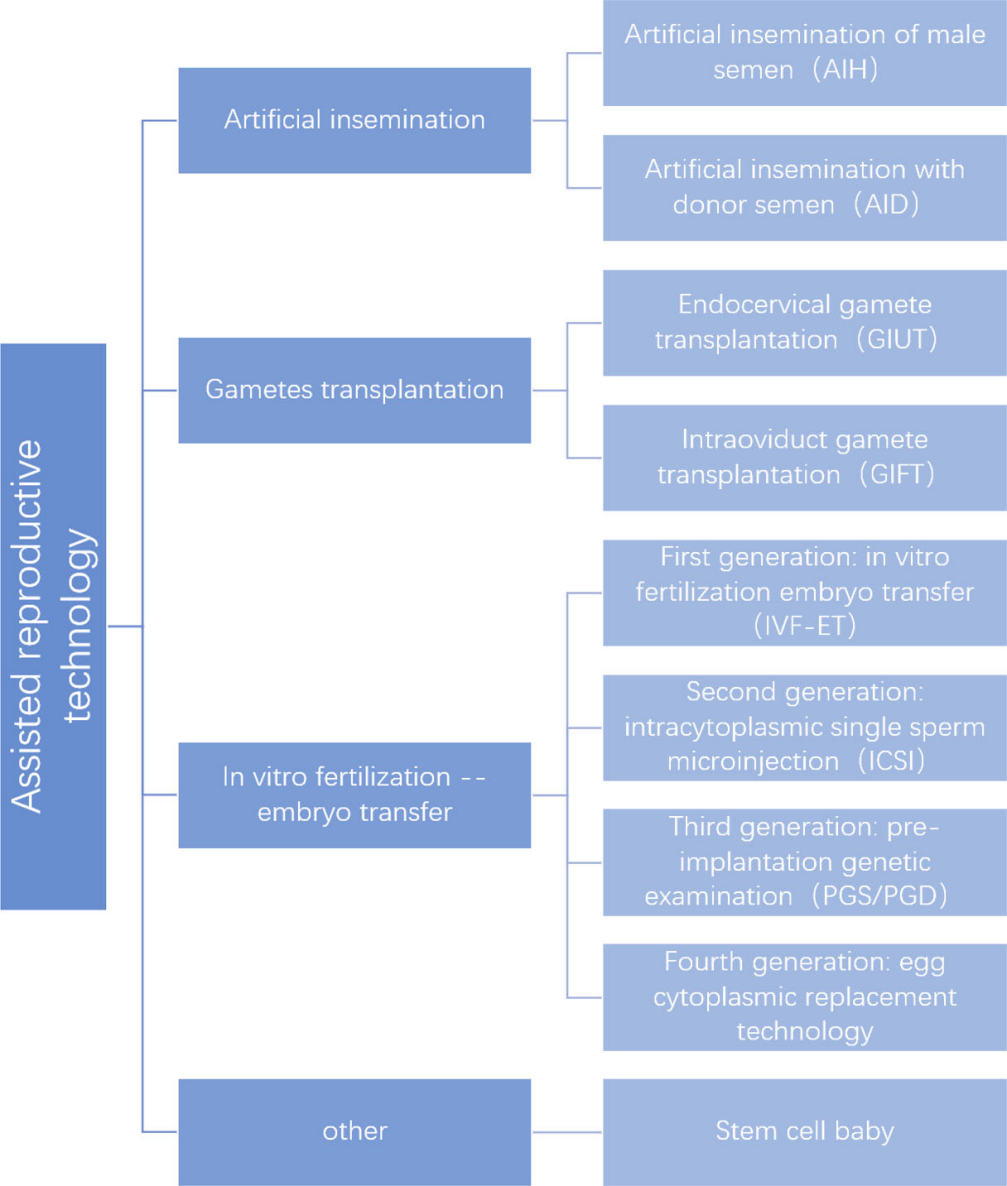
Assisted reproduction technology classification.

Many people opt for ART after trying medications and surgery for organic causes. At present, ART has become the primary method of infertility treatment. However, assisted reproduction is a medical vertical segmentation industry that has a relatively low degree of marketization and maturity throughout the medical industry. Since the first IVF birth in 1978 (44), ART has been used in the clinic for more than 40 years. ART has made an outstanding contribution to improving fertility outcomes and increasing birth rates worldwide with a series of developments (**Figure 1**).

According to Qiu et al., the costs of infertility treatment were mainly concentrated in “30,000–200,000 yuan”. Specifically, “50,000–100,000” accounted for 31.2%. In addition, 93.3% of patients said that the treatment had impacted the family finances to some degree, of which 17.6% took on debt. Moreover, 31.1% of patients claimed that their daily expense was greatly reduced (33, 34). Furthermore, in a notice on regulating and adjusting the prices of some medical services, Beijing regulated the prices of 16 assisted reproductive technologies, including 2,400 yuan for vaginal oocyte collection, 3,346 yuan for intracytoplasmic sperm injection 1,287 yuan for intrauterine artificial insemination, and 2,300 yuan for embryo transfer. Sperm separation resuscitation, selection, and centrifugation cost an average of 800 yuan per type, and *in vitro* fertilization–embryo culture need 1,566 yuan per day. The whole process is expensive, and if the parents have a single gene disease and need embryo testing, it will cost at least 6,000 to 7,000 yuan more.

Promoting the positive interactions among the medical insurance policy, price policy, medical service supply system, and payment mechanism can play the incentive and constraint role of the medical insurance payment on the professional behavior of doctors. This could result in accelerating the establishment of compatible resource allocation mechanisms among medical service providers, demanders, and medical insurance providers (45).

### Challenges for assisted reproduction centers and technology

The reproductive center integrates clinical and scientific research and teaching. Due to the fierce competition in the medical market, the legal system of assisted reproduction is not perfect. At present, there are still some illegal assisted reproduction institutions, malpractice by clinicians, and inappropriate use of new techniques. To tackle these threats, establishing a robust regulatory framework is an urgent need (46).

At present, ART and its application still have some controversial and unclear aspects. Impaired pregnancy outcomes in women using ART may be related to thin endometrium, especially on ovulation triggering days. Thin endometrium may raise the risk of female hypertensive disorders during pregnancy and infants who are small for their gestational age or with decreased birth weight (47). For embryo transfer patients with impaired ovarian reserve, the anti-mullerian hormone cannot predict clinical pregnancy (48). Paternal childbearing age as well as the use of ART influences the later generations’ bone mineral density (49). Recurrent implantation failure is very challenging in the clinical application of ART. Certain lymphocytes in serum have been identified as potential biomarkers, but further research is needed.

### The development status of ART in various countries worldwide

While the use of ART is increasing globally, there are wide disparities in access to treatment among different countries. It is estimated that at least 180 million couples suffered from infertility in developing countries (50). Daar and Merali divided the consequences of infertility and childlessness into seven levels——only a few developing countries will reach the third level (social violence and social isolation). It is estimated that access to IVF procedures is 100% in Belgium, while it is 1.1% in Africa (50). However, in developed countries, the situation is much more advanced—for example, in Finland, private clinics can provide over 60% of IVF treatments (51). Live birth rate per cycle is highest in the USA (28.4%), followed by Canada (23.9%), UK (22.8%), Scandinavia (21.5%), Japan (13.7%), and Australia (17.6%). While multiple birth rate per delivery in the USA is also the highest (34.2%), Japan has the lowest multiple pregnancy rate of the six countries covered (17%) (52). Denmark has the highest ART cycles per million in 14 countries across Europe. The average number of ART in Europe is 1,022 per million, while that of the USA and Israel is 395 and 3,000 in 2003, respectively (46).

The number of ART units varies considerably among different countries, and some factors like GDP are thought to have a close relationship with it. There are two to three ART units per million in Scandinavian countries, one to 1.5 in Western European countries, and less than one in the former Soviet Union (46). For the patients’ best interests, some doctors would recommend some cross-border reproductive facilities for the couples’ need (53).

### Reimbursement of infertility in various countries worldwide

The cost per live birth of IVF was highest in the USA and the UK ($41,132 and $40,364, respectively) and lowest in Scandinavia and Japan ($24,485 and $24,329, respectively) (52). The financial burden for the patients in Belgium was minimal based on the reimbursement policy of six IVF cycles per patient (50). The American Society of Reproductive Medicine reported that the average cost for a process of IVF is $12,400.18 in the USA (54), $8,500 in Canada, $6,534 in the UK, $5,645 in Australia, $5,549 in Scandinavia, and $3,956 in Japan (52). Smith et al. found that the average cost was $30,274 for IVF treatment and $7,704 for intrauterine insemination at a single institution. Besides this, women who received only medications were reported to have charges of $1,403 (55). In male infertility treatment, 64% of the out-of-pocket expenses are more than $15,000, whereas 16% are over $50,000 (56).

In addition, reimbursement for infertility treatment has become a state-mandated insurance coverage in the USA. Arkansas, Maryland, and Rhode Island offered a range of up to $15,000, $100,000, and $100,000 over a lifetime. In Hawaii, Connecticut, New Jersey, and Illinois, reimbursement is good for one, two, four, and six IVF cycles, respectively. Massachusetts, Montana, New York, Ohio, and West Virginia did not set a maximum reimbursement rate or limit the number of IVF cycles (54), but it is worth noting that, when examining IVF results, states with reimbursement were found to have lower rates of live births per cycle (57). Moreover, in any country, the total cost of IVF did not exceed 0.25% of healthcare expenditure (52). Furthermore, ART legislation also varied considerably across Europe (20). National health insurance covered 60% of doctors’ fees with part of examinations, and 50% of the drug costs were reimbursed by the Social Insurance Institution in Finland (51). Since the German healthcare modernization law was introduced in 2004, which induced the original 100% coverage for up to four cycles, no extensive age restriction has been cut to 50% reimbursement limited to three cycles and strict age limitation (46).

## Feasibility analysis of incorporating infertility into medical insurance

### Improve patients’ compliance

As a fundamental civil right, the reproductive right should be guaranteed. A survey by Merck found that nearly half of the patients who went to reproductive centers had been treated by other departments for 3 years. The time wasted in this process is very critical for women who intended to get pregnant (44). Notably, the acceptance of ART by the public affects the demand for ART in the public (58). If infertility is included in the coverage of medical insurance reimbursement, it will not only reduce the financial and psychological burden of patients but also effectively guide patients to medical insurance designated units for formal long-term treatment, which may reduce the unscrupulous advertising of criminal elements. In addition, it can encourage the patients to adhere to the same hospital for their continuous treatments and to reduce the duplications of medical resources, which can improve birth outcomes (40).

### Affordability of health insurance funds

**Figure 2:**
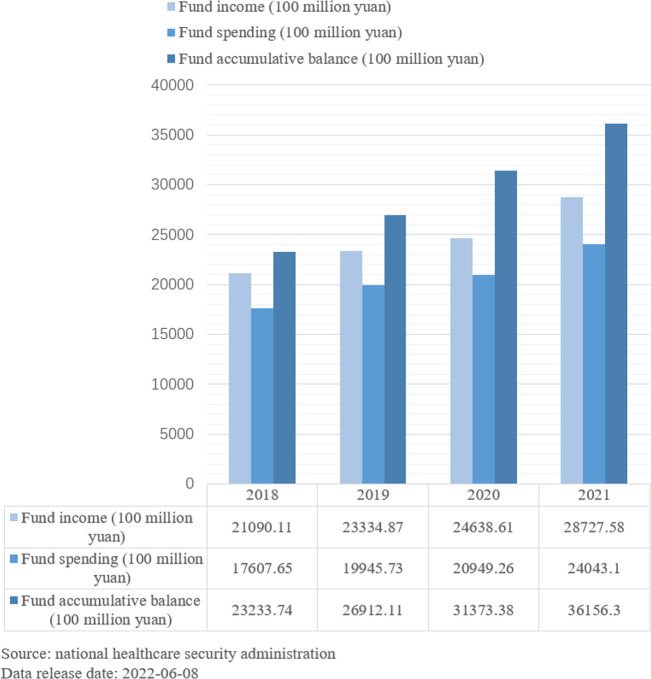
Income and expenditure of basic medical insurance fund, 2018–2021.

As shown in the chart (**Figure 2**), the affordability of the medical insurance fund is limited in China. At the same time, the financing of medical insurance is gradually expanding, and the surplus fund is abundant. The inclusion of infertility treatment in health insurance is debatable, and the burden on the health insurance fund could be potentially reduced by lowering the reimbursement rate or reducing the gross margin of treatment through market competition.

### Consideration of the economic burden of indirect diseases

The resources of assisted reproductive services are unevenly distributed, with sufficient resources in eastern provinces and insufficient resources in the central and western regions of China. Considering the large proportion of patients living in remote areas or rural areas with inconvenient transportation and undeveloped medical technology, they have to spend a lot of time and money on transportation and accommodation. Therefore, it is necessary to discuss whether appropriate transportation and accommodation subsidies would be taken into consideration for reimbursement through civil affairs assistance.

## Discussion and suggestions

### Selective reimbursement and giving preference to vulnerable groups

Belgium provides reimbursement for up to six IVF cycles per patient. In the United States, each state has several IVF cycles or a lifetime maximum reimbursement, and Germany has coverage for an age limit of four cycles. China is entering a depth of aging society dramatically, with an up-trend financial burden continually. According to the forecast, under the precondition of the current healthcare system, the balance of income and expenditure of China’s medical insurance fund would be hard to keep steady. In 2026, the annual balance will fall short for the first time, and in 2034, the cumulative balance will fall short for the first time (59). The increasing prevalence of infertility, coupled with the aging trend in China, has increased the need for ART. Affordability is the most essential consideration of the medical insurance system. At the current level of health insurance burden, it is possible to selectively cover some programs most needed by patients. The medical insurance reimbursement level of infertility patients should also be at a magnitude within the affordable range of medical insurance funds. As long as it does not affect the existing Medicare claims, the examination and treatment of infertility patients should be actively supported to intervene in the fertility rate effectively in the whole nation. For the main function of insurance, it is well recognized and highly recommended that medical insurance should favor vulnerable groups, such as poor and elderly couples who lost their only child. We can learn the advantage of the insurance for severe disease formation, with no minimum payment line and improved reimbursement level, of which corresponding medical assistance could be set up for application aggressively. Under the applicable circumstance, it is suggested that reduction or control of the out-of-pocket costs of poor patients to the acceptable range that would highlight the significant role of medical insurance be tried. Meanwhile, optimization of the structure of fund usage and appropriate consideration of the compensation of indirect costs could also benefit the patients who belong to the priority group with medical insurance support needed.

### Some commercial insurance covering infertility and assisted reproduction

The new medical reform plan should continually develop diverse plans of commercial health insurance, as commercial health insurance plays an essential role in the national medical security system. At present, commercial insurance for infertility and assisted reproduction remains limited; it requires more support from national policies to improve the situation by balancing the market related to the supply and demand among the whole society.

### Improve the technical skills of infertility treatment in China

The live birth rate per cycle is the highest in the USA, and the multiple pregnancy rate is relatively lower. The development of ART in China is relatively late and immature, and the success rate of ART is not high. Multiple pregnancies and other defects are still common. According to the assessment of ART in Liaoning Province from 2012 to 2016, the clinical pregnancy rate under the therapy of ART was only 45.59–53.63% (60). This means that, even under the circumstance with the assistance from relatively mature ART, nearly half of infertility patients are still unable to achieve their desire to have children (43). By the end of June 2020, there have been 523 medical institutions approved to carry out human-assisted reproduction technology and 27 medical institutions backed to set up human sperm banks (**Table 1**) in China. Only 396 hospitals were licensed for IVF, and 23.40% of the fertility centers could not meet the requirements of IVF technology. Due to the regional factor and lower population economic levels, the number of medical institutions is far less developed in western China compared to those located in eastern China. This imbalance of distribution could make it hard for infertility patients from economically underdeveloped areas to meet their family reproductive aspirations. Therefore, it would be better to encourage the medical institutions in the eastern region to help with the technical implementation and development in the western region by rotations or remote services to alleviate the uneven distribution of health resources and services. Besides this, the reality of assisted reproduction equipment and drugs in China is highly dependent on imports; therefore, it is under high warranty to develop assisted reproductive medical devices and related medication research.

**Table 1:** Number of reproductive center gaps by provinces and cities in China in the second half of 2020 (unit: per).

	1	2	3	4	5	6	7	8	9	10	11	12	13	14	15	16	17	18	19	20	21	22	23	24	25	26	27	28	29	30	31
a	56	33	31	31	31	30	27	23	21	19	18	18	18	17	15	14	13	13	12	12	10	10	9	9	8	8	8	4	2	2	1
b	54	40	33	30	30	30	27	28	20	17	18	16	21	20	19	20	16	13	13	15	12	9	10	8	10	6	4	6	1	2	2
c	-2	7	2	-1	-1	0	0	5	-1	-2	0	-2	3	3	4	6	3	0	1	3	2	-1	1	-1	2	-2	-4	2	-1	0	1

a, number of reproductive centers in the first half of 2020; b, planned number for 2020; c, gap; 1–31, Guangdong, Jiangsu, Shandong, Hebei, Henan, Hubei, Zhejiang, Hunan, Guangxi, Shanghai, Liaoning, Jiangxi, Beijing, Yunnan, Fujian, Anhui, Sichuan, Guizhou, Shanxi, Tianjin, Heilongjiang, Chongqing, Shanxi, Jilin, Neimenggu, Hainan, Xinjiang, Gansu, Qinghai, Ningxia, and Xizang (source: National Health Commission).

What is worth paying attention to is that, in 2005, Hansen et al. analyzed the results of 25 related studies and reported that the incidence of birth defects in ART offspring increased by 25% or more compared to the rate in a natural pregnancy (61). The increase in birth defects caused by ART should be addressed by improved technology.

### Regulate the application of ART

With the continuous development of ART technology, more attention should be paid to the modifications and revisions of relevant regulations. Local governments would help with the implementation of regulations by gradually introducing local regulations and supplementing their policies with special campaigns to encourage or regulate the use of ART in qualified medical institutions. Other issues related to ART might exist in the gray—like egg donation and surrogacy; thus, stricter regulation and legal-level control are also urgently required.

In our modern society with diverse healthcare systems, insurance plays a critical role in ensuring the patients’ authority for suitable healthcare and minimizing the related financial burden. Although the examination and treatment of infertility are still far behind the level of full coverage by medical insurance around the whole nation of China at present, there is a huge potential to improve patients’ compliance and promote the development and implementation of ART through the selective inclusion of medical insurance reimbursement and policies focusing on the vulnerable groups. In response to Suggestion No. 5581 of the Fourth Session of the 13th National People’s Congress, the medical insurance department in China has announced that some eligible fertility support drugs, including bromocriptine, triptorelin, clomiphene, and other ovulation promotion drugs, will be included into medical insurance at the scope of a rational price to improve the Medicare and medication services of infertility patients. In addition, without affecting the existing circumstances of current affordable medical insurance funds, different support measures are further developed—for example, 16 mature, safe, and reliable ART items, such as intrauterine insemination technology, embryo transfer, and sperm selection, which are common in outpatient clinics, will be included in category A of medical insurance reimbursement at 15 designated medical facilities in Beijing this year. With the increase being in public attention, it is believed that the reproductive rights of infertile patients will be under a fast-developing protection pathway in China.

### Raise male awareness of infertility

A large proportion of male patients said that they had only discussed their infertility with their wives and felt uncomfortable or even ashamed of talking about it. For a long time, from the bottom of their hearts, most people have believed that women are most responsible for infertility (62), and women have been blamed more and received more useless tests and even treatments. Male factors must be taken into account in the early stages of a fertility assessment, as the minor problems of male infertility can be treated with appropriate drugs to relieve the economic, psychological, and physical stress on both partners. Raising the level of awareness of male infertility is now vital. Through the examination of male infertility, we can achieve timely prediction and prevention of cardiovascular diseases, occurrence or development chronic comorbidities, and mortality and improve the overall male health survival. We can gradually and comprehensively raise people’s awareness of male infertility and improve men’s awareness of going to a hospital for examination through many ways—classroom education in schools, popularization in the process of routine physical examination and medical treatment in hospitals, and publicity in communities (7).

## Author contributions

LW proposed the central idea and is responsible for the main writing and subsequent revision of the paper. XX and TW also participated in writing the first draft of the paper. YZ analyzed most of the data. QT and JL contributed to the elaboration and additional analysis. YW and WH reviewed and revised the article many times. WW provides financial support. WW is responsible for reviewing the final manuscript. All authors contributed to the article and approved the submitted version.

## Funding

This work was supported by the Key Laboratory of National Health Commission and Jiangsu Provincial Health Development Research Center Open Subject in 2021 (JSHD2021047), Health Jiangsu Research Institute 2022 Annual Decision-Making Consultation and Cultivation Project, and the Priority Academic Program for the Development of Jiangsu Higher Education Institutions (Public Health and Preventive Medicine).

## Acknowledgments

Our deepest gratitude goes first and foremost to Professor Wu for his constant encouragement and guidance. Without his consistent and illuminating instruction, this paper could not have reached its present form. Then, we would like to thank our colleagues for writing, revising, and organizing the paper.

## Conflict of interest

The authors declare that the research was conducted in the absence of any commercial or financial relationships that could be construed as a potential conflict of interest.

## Publisher’s note

All claims expressed in this article are solely those of the authors and do not necessarily represent those of their affiliated organizations, or those of the publisher, the editors and the reviewers. Any product that may be evaluated in this article, or claim that may be made by its manufacturer, is not guaranteed or endorsed by the publisher.

## Abbreviations

ART, assisted reproductive technology; IVF, *in vitro* fertilization; IVF-ET, *in vitro* fertilization and embryo transfer; ICSI, intracytoplasmic sperm injection; IUI, intrauterine insemination.
